# Supplementary material for: Associations of social determinants of health and patient safety in perinatal care: Protocol for a systematic review with meta-analysis
Source: PLoS One. 2025 Nov 4;20(11):e0336013. doi: 10.1371/journal.pone.0336013 (PMC12585019; doi:10.1371/journal.pone.0336013)
Supplement: S1 Checklist — SDoH and perinatal patient safety. (DOC) [file pone.0336013.s001.doc]

**Supporting Information**

**S1 PRISMA-P Checklist for Manuscript entitled ‘Association of social determinants of health and patient safety in perinatal care: Protocol for a systematic review with meta-analysis’**

| Section and topic | Item No | Checklist item |
| --- | --- | --- |
| ADMINISTRATIVE INFORMATION | | |
| Title: |  |  |
| Identification | 1a | Association of social determinants of health and patient safety in perinatal care: Protocol for a systematic review with meta-analysis |
| Update | 1b | n/a |
| Registration | 2 | PROSPERO: [CRD420251090149](https://www.crd.york.ac.uk/PROSPERO/view/CRD420251090149)  OSF: [doi.org/10.17605/OSF.IO/UP3JS](https://doi.org/10.17605/OSF.IO/UP3JS) |
| Authors: |  |  |
| Contact | 3a | Katharina Averdunk ¹, Céline Miani ², Brigitte Strizek ³, Matthias Weigl ¹  ¹ Institute for Patient Safety (IfPS), Medical Faculty, University Hospital Bonn, Bonn, Germany  ² Department of Epidemiology and International Public Health, School of Public Health, Bielefeld University, Bielefeld, Germany  ³ Department of Obstetrics and Prenatal Medicine, University Hospital Bonn, Bonn, Germany  Corresponding author: Katharina Averdunk M.Sc., Institute for Patient Safety (IfPS), University Hospital Bonn, Venusberg-Campus 1, 53127 Bonn, Germany, katharina.ahring-averdunk@ukbonn.de |
| Contributions | 3b | KA: Guarantor, conceptualisation, methodology, writing – original draft preparation CM, BS: Methodology, writing – review and editing MW: Conceptualisation, methodology, supervision, writing – review and editing |
| Amendments | 4 | n/a |
| Support: |  |  |
| Sources | 5a | The authors received no specific funding for the research. The publication was supported by the Open Access Publication Fund of the University of Bonn. |
| Sponsor | 5b | n/a |
| Role of sponsor  or funder | 5c | n/a |
| INTRODUCTION | | |
| Rationale | 6 | A plethora of studies suggest adverse outcomes among women and neonates in disadvantaged conditions. Nevertheless, the extant evidence base on the specific ramifications of patient safety within adverse perinatal outcomes remains ambiguous. This lack of evidence implies substantial challenges for advanced research designs, the assessment of care quality and safety, and the development of targeted interventions. |
| Objectives | 7 | To outline definitions and methods that will be used to synthesise and integrate international evidence on associations between social determinants of health and patient safety in perinatal care. |
| METHODS | | |
| Eligibility criteria | 8 | Study design: Original studies reporting on associations between social determinants of health and perinatal patient safety (Quantitative observational studies, including retrospective and prospective, cross-sectional and longitudinal designs, cohort and case-control studies, randomised controlled trials).  Population: Patients during their pregnancy, childbirth and the puerperium, as well as their foetuses and neonates.  Exposure: Patient characteristics, captured in the concept of ‘social determinants of health’; exclusive medical conditions, e.g., chronic or severe diseases, mental health, substance abuse, STD, disabilities.  Outcome: Any subjective or objective safety outcome, if the specific aim was to empirically investigate perinatal patient safety.  Setting: Antepartum, intrapartum, postpartum and neonatal care. |
| Information sources | 9 | Literature search in MEDLINE (via PubMed), Scopus database, CINAHL (via EBSCO), and Embase (via Elsevier). Forward and backward citation tracing and snowballing techniques among similar systematic reviews. |
| Search strategy | 10 | (("pregnant people"[MeSH Terms] OR "pregnancy"[MeSH Terms] OR "peripartum period"[MeSH Terms] OR "postpartum period"[MeSH Terms] OR "obstetric"[Title/Abstract] OR "perinatal"[Title/Abstract] OR "gestation*"[Title/Abstract] OR "pregnan*"[Title/Abstract] OR "prepartum"[Title/Abstract] OR "antenatal"[Title/Abstract] OR "antepartum"[Title/Abstract] OR "prenatal"[Title/Abstract] OR "parturi*"[Title/Abstract] OR "peripartum"[Title/Abstract] OR "intrapartum"[Title/Abstract] OR "intranatal"[Title/Abstract] OR "labor"[Title/Abstract] OR "labour"[Title/Abstract] OR "postnatal"[Title/Abstract] OR "puerper*"[Title/Abstract] OR "postpartum"[Title/Abstract]) AND ("social determinants of health"[MeSH Terms] OR "socioeconomic factors"[MeSH Terms] OR "unemployment"[MeSH Terms] OR "poverty"[MeSH Terms] OR "educational status"[MeSH Terms] OR "literacy"[MeSH Terms] OR "communication barriers"[MeSH Terms] OR "health services accessibility"[MeSH Terms] OR "insurance coverage"[MeSH Terms] OR "social conditions"[MeSH Terms] OR "social vulnerability"[MeSH Terms] OR "violence"[MeSH Terms] OR "social discrimination"[MeSH Terms] OR "refugees"[MeSH Terms] OR "transients and migrants"[MeSH Terms] OR "incarceration"[MeSH Terms] OR "sex workers"[MeSH Terms] OR "racism"[MeSH Terms] OR "food insecurity"[MeSH Terms] OR "ethnicity"[MeSH Terms] OR "war exposure"[MeSH Terms] OR "religion"[MeSH Terms] OR "determinants of health"[Title/Abstract] OR "socioeconomic factor*"[Title/Abstract] OR "socio-economic factor*"[Title/Abstract] OR "socioeconomic condition*"[Title/Abstract] OR "socio-economic condition*"[Title/Abstract] OR "socioeconomic status"[Title/Abstract] OR "socio-economic status"[Title/Abstract] OR "socioeconomic characteristic*"[Title/Abstract] OR "socio-economic characteristic*"[Title/Abstract] OR "social condition*"[Title/Abstract] OR "economic factor*"[Title/Abstract] OR "vulnerability"[Title/Abstract] OR "low income"[Title/Abstract] OR "poverty"[Title/Abstract] OR "unemploy*"[Title/Abstract] OR "educational status"[Title/Abstract] OR "educational level"[Title/Abstract] OR "communication barrier*"[Title/Abstract] OR "language barrier*"[Title/Abstract] OR "illitera*"[Title/Abstract] OR "health services accessibilit*"[Title/Abstract] OR "access to healthcare"[Title/Abstract] OR "access to health services"[Title/Abstract] OR "access to care"[Title/Abstract] OR "insurance coverage"[Title/Abstract] OR "insurance status"[Title/Abstract] OR "housing instabilit*"[Title/Abstract] OR "housing insecurit*"[Title/Abstract] OR "homeless*"[Title/Abstract] OR "poor housing"[Title/Abstract] OR "incarerat*"[Title/Abstract] OR "imprison*"[Title/Abstract] OR "sex worker*"[Title/Abstract] OR "prostitute*"[Title/Abstract] OR "discriminat*"[Title/Abstract] OR "raci*"[Title/Abstract] OR "ethnic*"[Title/Abstract] OR "race"[Title/Abstract] OR "refugee"[Title/Abstract] OR "migrant"[Title/Abstract] OR "migrat*"[Title/Abstract] OR "asylum"[Title/Abstract] OR "displaced"[Title/Abstract] OR OR "violence"[Title/Abstract] OR "food insecurit*"[Title/Abstract] OR "water insecurit*"[Title/Abstract] OR "adolescen*"[Title/Abstract] OR "gender identity"[Title/Abstract] OR "war exposure"[Title/Abstract] OR "religion*"[Title/Abstract] OR "religious belief*"[Title/Abstract]) AND ("patient safety"[MeSH Terms] OR "patient harm"[MeSH Terms] OR "medical errors"[MeSH Terms] OR "treatment failure"[MeSH Terms] OR "patient safety"[Title/Abstract] OR "safety of care"[Title/Abstract] OR "inpatient safety"[Title/Abstract] OR "unsafe care"[Title/Abstract] OR "unsafe healthcare"[Title/Abstract] OR "patient harm*"[Title/Abstract] OR "near miss*"[Title/Abstract] OR "close call*"[Title/Abstract] OR "medical error*"[Title/Abstract] OR "medical mistake*"[Title/Abstract] OR "critical incident*"[Title/Abstract] OR "never event*"[Title/Abstract] OR "medication error*"[Title/Abstract] OR "harmful incident*"[Title/Abstract] OR "safety outcome*"[Title/Abstract] OR "safety incident*"[Title/Abstract] OR "safety event*"[Title/Abstract] OR "adverse event*"[Title/Abstract] OR "adverse outcome*"[Title/Abstract] OR "experienced safety"[Title/Abstract] OR "perceived safety"[Title/Abstract] OR "reported safety"[Title/Abstract] OR "treatment failure*"[Title/Abstract] OR "safety behavior"[Title/Abstract] OR "safety behaviour"[Title/Abstract])) |
| Study records: |  |  |
| Data management | 11a | Electronic data management system (Rayyan) for facilitating collaboration among reviewers. |
| Selection process | 11b | Level 1 (title/abstract) and level 2 (full text) screening of literature search results, conducted by two independent and trained reviewers, using a standardised documentation form informed by eligibility criteria. |
| Data collection  process | 11c | Data extraction from eligible studies will be performed independently and in duplicate, using a standardised form informed by eligibility criteria. All procedures will be piloted in a subset of studies. |
| Data items | 12 | Study design: Design, location (country), health care setting (inpatient/ outpatient), perinatal care setting (antepartum, intrapartum, postpartum, neonatal), study population (patient group(s), sample size, special characteristics), sampling method, level of analysis (individual, population-/ group-based), source of financial support, and special considerations, e.g., study related to COVID-19 pandemic.  Population: Stage of pregnancy, childbirth or puerperium of included individuals and/ or study groups.  Exposure: Reported social determinants of health. Classification into subdomains of social determinants of health: Economic stability, education access and quality, health care access and quality, neighbourhood and built environment, social and community context, individual constitutional factors, and refugee status.  Outcome: Any reported quantitative data and measures pertaining to perinatal patient safety. Classification into types and causes of patient safety constraints, including a category ‘not classifiable’. Additional classification according to data sources such as self-reported, clinical data, or patient record data will be considered.  For each exposure condition and outcome, data sources, types of data, measurement tools, summary statistics, and confounders will be extracted. Furthermore, if reported, result data (measures of association), and effect sizes will be extracted. |
| Outcomes and prioritization | 13 | Estimates on associations between SDoH and perinatal patient safety on a study- and an individual patient level. Expected challenges regarding variation in exposure and outcome measures will be addressed by assessing associations between clustered variables and outcomes. We will determine associations using correlation coefficients appropriate to each type of data. |
| Risk of bias in individual studies | 14 | Methodological quality and risk of bias of eligible studies will be assessed using the critical appraisal tools proposed by Joanna Briggs Institute (JBI). Each of the domains will be judged as to the possible risk of bias, and will be rated as ‘high risk’ and ‘low risk’. If essential information concerning one or more domains is missing, risk of bias will be rated as ‘unclear’. Two authors will review risk of bias independently. Reviewers will not be blind to the studies. |
| Data synthesis | 15a | Quantitative synthesis will be conducted if deemed appropriate based on assessment of clinical, methodological, and statistical heterogeneity. |
|  | 15b | I. Clinical and methodological heterogeneity: Narrative data synthesis, using structured tables to present and compare study and patient characteristics, reported measures, and key findings. Variations will be discussed with respect to their potential impact on pooling data and review findings.  II. Statistical heterogeneity: Cochran’s Q and I² tests. Statistically significant heterogeneity will be indicated if Cochran’s Q is large (to be interpreted depending on the number of included studies), or if I ²≥ 50% or p < 0.1. In such cases, conducting meta-analyses of the entire set of studies may not be appropriate. Instead, the strength of individual effects will be presented visually via harvest plots. Additional meta-analyses according to exposure and outcome groups will be considered. |
|  | 15c | Subgroup analyses according to the following criteria: Stage of pregnancy, perinatal care settings, and countries’ income-level. Additional meta-regression methods, if deemed appropriate after data extraction and aggregation, and examining effects of cumulated exposure conditions. Sensitivity analyses by omitting studies that were judged to be at high risk of bias. |
|  | 15d | Descriptive summary statistics, narrative synthesis, and visual representation via harvest plots for comparing individual effects observed in included studies. |
| Meta-bias(es) | 16 | Assessment of publication bias through visual inspection of funnel plots and tests for asymmetry, if sufficient studies were included. |
| Confidence in cumulative evidence | 17 | Assessment of quality of evidence for all outcomes using the GRADE tool. |
